# Machine Learning‐Based Prediction of Determinants of Appropriate Complementary Feeding Practices Among Women With Children Aged 6–23 Months in Sub‐Saharan Africa

**DOI:** 10.1002/fsn3.70837

**Published:** 2025-08-27

**Authors:** Nebebe Demis Baykemagn, Winta Tesfaye, Hiwot Tezera Endale, Mihret Getnet, Tseganesh Asefa, Fethiya Seid Hasen, Destaye Tirite Gelaw, Yihun Tefera Ayenew, Tirualem Zeleke Yehuala, Alemu Teshale Bicha, Habtu Kifle Negash

**Affiliations:** ^1^ Department of Health Informatics, Institute of Public Health, College of Medicine and Health Sciences University of Gondar Gondar Ethiopia; ^2^ Department of Human Physiology, School of Medicine, College of Medicine and Health Sciences University of Gondar Gondar Ethiopia; ^3^ Department of Medical Biochemistry, School of Medicine, College of Medicine and Health Sciences University of Gondar Gondar Ethiopia; ^4^ Department of Epidemiology and Biostatistics, Institute of Public Health, College of Medicine and Health Sciences University of Gondar Gondar Ethiopia; ^5^ Department of Medical Nursing, School of Nursing, College of Medicine and Health Sciences University of Gondar Gondar Ethiopia; ^6^ Department of Human Anatomy, School of Medicine, College of Medicine and Health Sciences University of Gondar Gondar Ethiopia; ^7^ Department of Obstetrics and Gynecology, School of Medicine, College of Medicine and Health Sciences University of Gondar Gondar Ethiopia

**Keywords:** child, digital health, feeding practices, machine learning, nutrition, woman

## Abstract

Globally, children's feeding practices are a major public health concern. Evidence indicates that 90% of children receive less than the bare minimum of dietary content. In developing countries, only one out of five children under 24 months old receives the minimum recommended diet. This study utilized a weighted dataset of 24,235 from the Demographic and Health Survey (DHS) conducted across eight Sub‐Saharan African countries. The data preprocessing and analysis were performed using STATA version 17 and Python 3.10. Feature scaling was carried out using MinMax scaling and Standard Scalar, while feature selection was performed through Recursive Feature Elimination (RFE). An 80:20 data split ratio was applied, and class imbalance was addressed using Tomek Links combined with Random Oversampling. Eight models were selected and trained with both balanced and unbalanced datasets. Model performance was evaluated using metrics such as ROC‐AUC, accuracy, and the confusion matrix. The overall current feeding practice rate is 9.1%, with a Random Forest model achieving an accuracy of 91% and an AUC of 96%. The predictors for appropriate complementary feeding include current breastfeeding status, maternal education, wealth status, number of household members, sex of the household head, sex of the child, place of delivery, maternal employment status, and distance to the nearest health facility. In conclusion, the Random Forest model was effectively used to identify key determinants, revealing that appropriate complementary feeding practices are low. To improve this, we recommend enhancing community‐based nutrition and reproductive health education, providing economic support for the poor, increasing access to healthcare facilities, and creating better opportunities to access smartphones and other social media platforms.

## Graphical abstract

This study analyzed a weighted DHS dataset of 24,235 from eight Sub‐Saharan African countries using STATA and Python. Features were scaled and selected via RFE. After an 80:20 split, class imbalance was handled with Tomek Links and oversampling. Eight models were trained and evaluated using ROC‐AUC, accuracy, and confusion matrix. Key predictors of complementary feeding included current breastfeeding status, maternal education, wealth, household size, child's sex, place of delivery, maternal employment, and healthcare access. Recommendations focus on nutrition education, economic support, and improved healthcare and technology access.

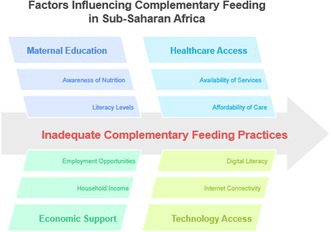

## 1 Introduction

Globally, children's feeding practices are a major public health concern (Dukhi 2020). Evidence indicates that 90% of children receive less than the bare minimum of dietary content (DevelopmentAid n.d.) In developing countries, just one out of five children under 24 months old gets the minimum recommended diet (Belay et al. 2022). These alarming findings are primarily the result of inadequate supplemental feeding practices. A child's optimal growth depends on adequate complementary food starting at 6 months of age, as this is a critical developmental period and when the majority of stunting cases occur in many countries (WHO n.d.‐a).

Complementary feeding is defined by the World Health Organization (WHO) as the “process of providing foods in addition to milk for children aged 6–23 months” (WHO 2023). A crucial phase in a child's growth is complementary feeding, especially for those between the ages of 6–23 months (Wu et al. 2024). This transition period, from exclusive breastfeeding to family foods, begins at 6 months when breast milk alone no longer meets a child's nutritional needs. Complementary feeding promotes physical, cognitive, and motor development, laying the foundation for future health and learning (WHO n.d.‐b).

More than 155 million children under five experience stunting, which contributes to over one million deaths, with 41% occurring in children under one year old (Takele et al. 2022). Following this, the WHO plans to achieve a 40% decrease in stunting prevalence by 2025 (Quamme and Iversen 2022). In sub‐Saharan African countries, where the prevalence of appropriate complementary feeding practices among mothers of children aged 6–23 months is only 13.02%, innovative strategies are needed to achieve the WHO's plan and reduce child morbidity and mortality (Mekonen et al. 2024).

Poor feeding practices combined with an inadequate amount and quality of supplementary foods (White et al. 2017) can lead to a range of negative outcomes in children, including vitamin deficiencies that affect brain development, bone health, and vision; weakened immunity; increased vulnerability to diarrhea; growth delays; long‐term health complications; and lasting social and economic consequences (Masuke et al. 2021; Ishaq et al. 2024; Liu and Chang 2023; Shastak and Pelletier 2024).

Here is the WHO recommendation in terms of time and frequency: Complementary foods should be introduced at 6 months, alongside breast milk. Initially, 2–3 times a day (6–8 months), increasing to 3–4 times daily from 9 to 24 months (WHO n.d.‐c). Moreover, adequate (including protein and micronutrients), safe (hygienically stored and prepared), and properly fed (with suitable frequency and feeding appropriate for age) are additional criteria (WHO n.d.‐d).

According to earlier studies, wealth index, access to health services, maternal employment, cultural beliefs and practices, place of residence, knowledge of nutrition, breastfeeding practices, health status of the child, health information, hygiene practices, duration of exclusive breastfeeding, attendance at postnatal care services, household food security, frequent antenatal care visits, institutional delivery, and the educational status of the household head are factors that influence supplemental feeding practices (Kiran et al. 2022; Mekonen 2025; Singh et al. 2017).

Machine learning (ML) offers sophisticated analytical approaches to manage vast data sets and find nonlinear correlations, while previous research has been carried out using traditional methods, which frequently rely on linear models that may not capture the complicated relationships with the outcome variable (Bhatnagar 2018). The aim of this study is to identify and predict significant features associated with feeding practices from a multi‐country dataset, using machine learning techniques. The findings aim to inform potential areas for future intervention and provide evidence‐based insights for policy considerations, particularly in low‐ and middle‐income country contexts.

## 2 Methods and Materials

### 2.1 Study Design and Setting

The study design is a cross‐sectional study based on secondary data. We utilized recent Demographic and Health Surveys (DHS) data from eight Sub‐Saharan African countries: Burkina Faso, Côte d'Ivoire, Ghana, Kenya, Lesotho, Madagascar, Mozambique, and Tanzania, collected between 2020 and 2023. Machine learning techniques were applied to identify and predict key features associated with feeding practices.

Sub‐Saharan Africa (SSA) refers to the region of the African continent located south of the Sahara Desert, home to over 1 billion people. This diverse and complex region encompasses several areas: West Africa, including countries like Nigeria, Ghana, Senegal, and Côte d'Ivoire; East Africa, which features countries such as Kenya, Ethiopia, Tanzania, and Uganda; Central Africa, with nations like the Democratic Republic of Congo, Cameroon, and Gabon; and Southern Africa, which includes South Africa, Botswana, Namibia, and Angola.

### 2.2 Source and Study Population

The study population consists of all mothers of children aged 6–23 months in Sub‐Saharan Africa, using data from the most recent DHS surveys conducted between 2020 and 2023.

### 2.3 Study Variables

*Dependent variable*: Complementary feeding practices (appropriate and inappropriate).

*Independent variables*: marital status, education status, social media use, wealth status, age of mother, place of delivery, smartphone availability, distance to healthcare facility (HF), residence, sex of the household (HH) head, number of household members, birth interval, current breastfeeding status, sex of the child, number of antenatal care (ANC) visits, postnatal care (PNC) check‐ups, child's age in months, and mother's work status.

### 2.4 Data Processing and Management

After deciding on the title by considering the current public health issue, extract data from the individual record dataset (IR). Then, review the relationships between all variables and the outcome based on previous study evidence and expert opinions. Check the similarity of the selected variables across four countries, namely Burkina Faso, Côte d'Ivoire, Ghana, Kenya, Lesotho, Madagascar, Mozambique, and Tanzania, that have recent DHS data available. This dataset is crucial for the current intervention and policy formulation. Finally, append the data from all four countries using Stata version 17. For data preprocessing, export to Python Colab version 3.10.2. First, check for missing values using the code dataset. Is null ().sum (). Then, drop the variables with more than 25% missing values. For variables with missing values below the 25% threshold, use Mode Imputation or K‐Nearest Neighbors (KNN) Imputation, depending on the nature of the features, especially for crucial outcome variables. After handling the missing values, extract the outcome variable independently from the independent variables.

### 2.5 Feature Engineering

Encoding Categorical Data: Recode variables based on the literature, use One‐Hot Encoding to prepare for easier further analysis.

Many machine learning algorithms are sensitive to the scale of features (Analytics Vidhya n.d.). Feature scaling is the process of bringing variables to a similar scale, and in this study, MinMaxScaler and Standard Scaler were applied. The goal is to minimize performance bias in models that are sensitive to feature scaling, as the absence of standardization can negatively impact model performance. Feature creation is another crucial aspect of feature engineering in this study, where a social media utilization variable was developed by summing the frequencies of radio, television, and newspaper usage.

### 2.6 Feature Selection

Recent studies indicate that irrelevant variables weaken the model's capacity for generalization, raise its overall complexity, and possibly lower a classifier's overall accuracy in machine learning study (Kirk et al. 2022). Recursive Feature Elimination (RFE) was applied iteratively and systematically to eliminate irrelevant features. It evaluates features in the context of others, reducing dimensionality while preserving the most predictive variables. This enhances model efficiency, improves generalizability, and helps reduce overfitting.

### 2.7 Splitting the Data

Previous research indicates that splitting data helps provide an accurate assessment of a model's performance on unseen data and aids in detecting overfitting (Muraina 2022). In this study, the data was split into an 80:20 ratio for training and testing to assess the model's performance and prevent overfitting in the machine learning algorithms. This was achieved using Train‐Test Split and Cross‐Validation (10‐fold validation) techniques.

### 2.8 Handling Imbalanced Data

In machine learning, class imbalance is a common issue, as uneven data distribution can significantly impact the accuracy of the model (Analytics Vidhya n.d.).

To tackle this issue, this study combined Tomek Links with Random Oversampling. This approach aims to enhance the model's generalization ability by ensuring fair representation of both classes and minimizing bias.

### 2.9 Model Selection

After thoroughly reviewing machine learning studies in feeding practice (Kirk et al. 2022; Ennaji et al. 2023) we selected seven supervised machine learning models for consideration: Decision Tree (DT), Random Forest (RF), k‐Nearest Neighbors (KNN), Artificial Neural Network (ANN), eXtreme Gradient Boosting (XGBoost), Light Gradient Boosting Machine (LGBM), Adaptive Boosting (AdaBoost), and Gradient Boosting (GB).

### 2.10 Model Training

Once the model was chosen, both balanced and unbalanced datasets were used to train the selected classifiers, with tenfold cross‐validation applied to assess their performance. The best‐performing predictive model was then identified through comparison and retrained using the balanced training data to make final predictions on unseen test data.

### 2.11 Model Evaluation

Recent studies have highlighted that model evaluation is an essential step in machine learning, as it enables us to assess how effectively a trained model performs on unseen data (Schröder and Schulz 2022; Parmezan et al. 2019).

**FIGURE 1:**
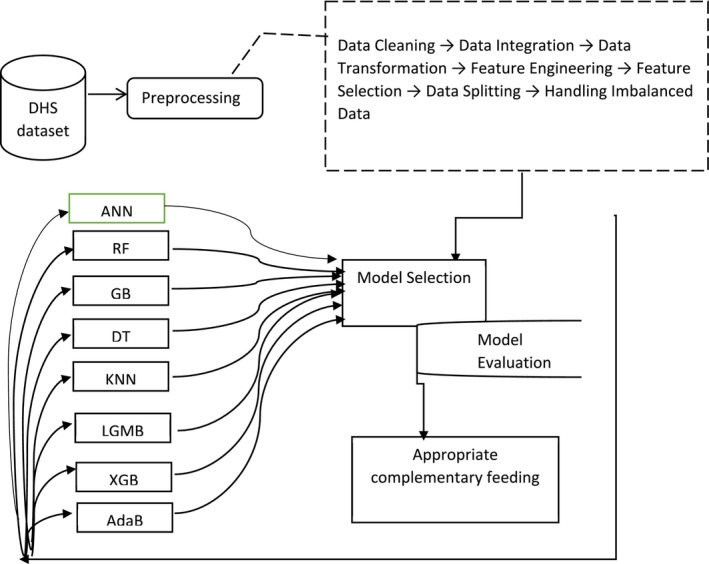
Data preprocessing and management procedures for appropriate complementary feeding.

Machine learning (ML) has shown great promise in the healthcare system, where large and complex datasets can provide valuable insights into various aspects, from patient management to policy formulation. However, with this potential comes the responsibility to carefully measure and guard against biases and inflation, in order to enhance healthcare service quality and generate robust evidence (Miller et al. 2024). To do this, F1‐Score, ROC‐AUC, Accuracy, Precision, and Recall were used in this study's model evaluation. The overall data preprocessing and management procedures are shown in Figure 1.

## 3 Results

### 3.1 Feature Selection

**FIGURE 2:**
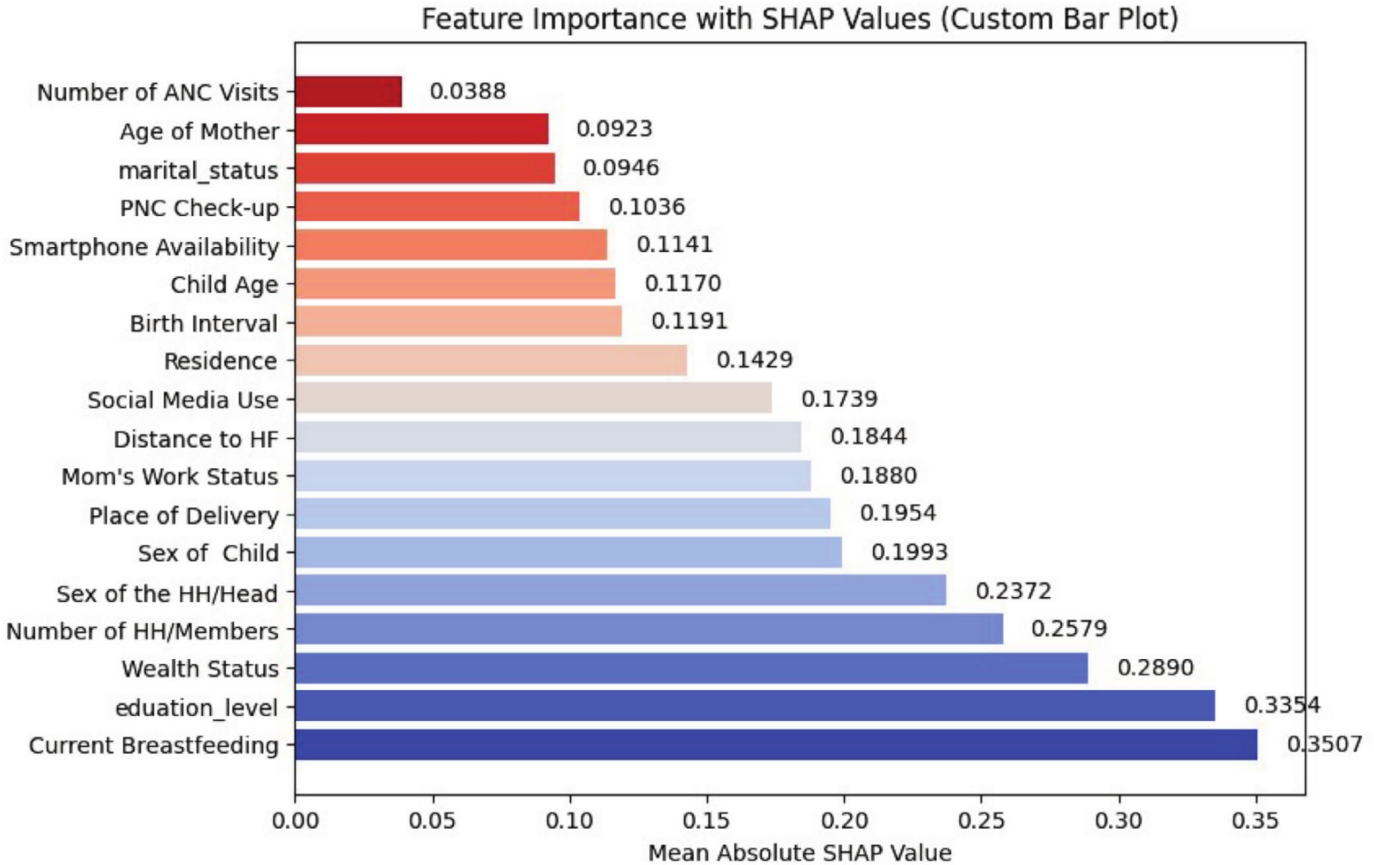
Feature importance for predicting complementary feeding practices.

The Recursive Feature Elimination (RFE) analysis showed that the following features are the most relevant for predicting appropriate complementary feeding practices among women with children aged 6–23 months (Figure 2).

### 3.2 Handling Imbalanced Data

**FIGURE 3:**
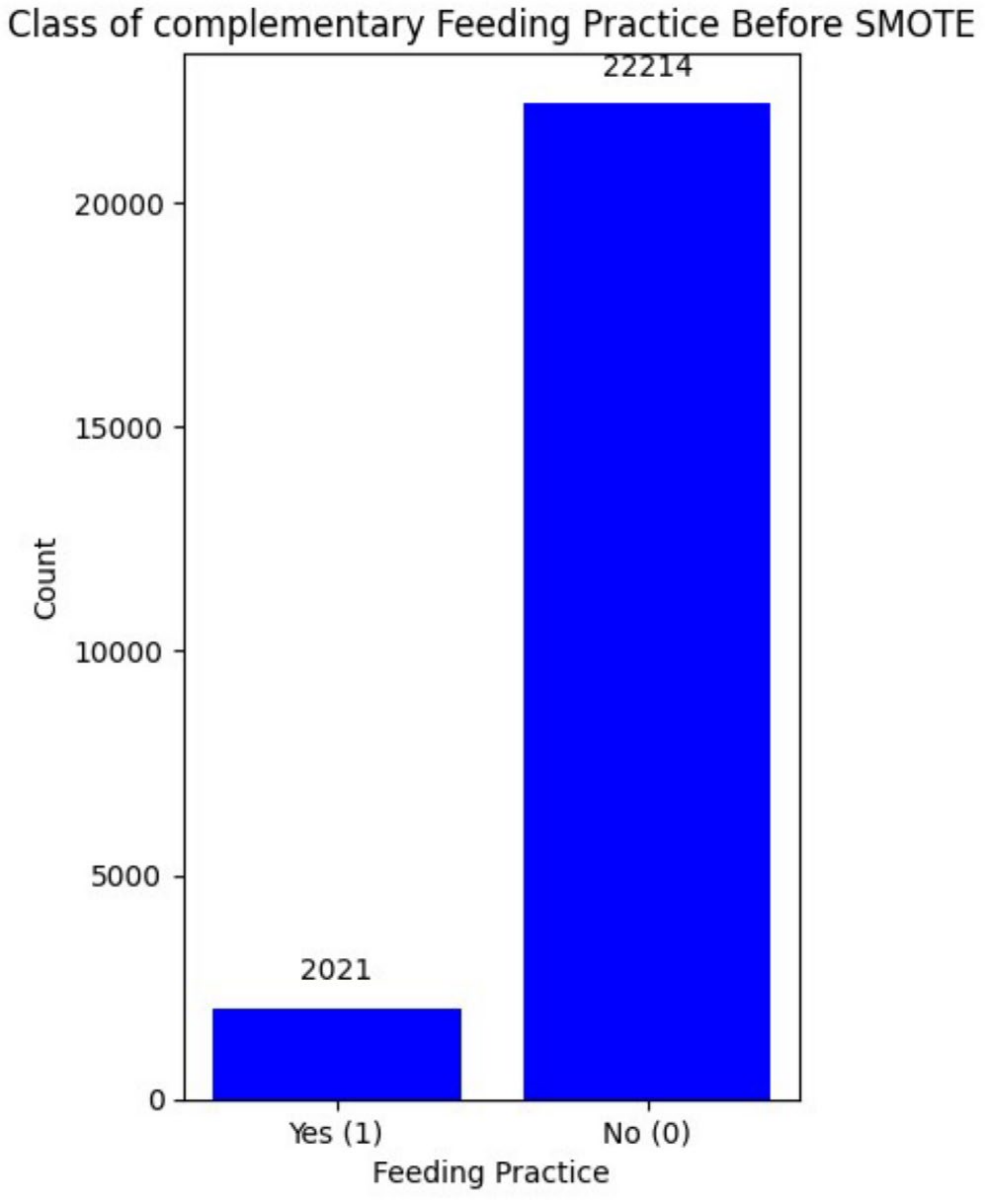
Class distribution before SMOTE.

The SMOTE analysis showed that before balancing the data, there were 2021 “Yes” cases and 22,214 “No” cases. After applying SMOTE, both classes were balanced to 22,214 each (Figure 3).

### 3.3 Complementary Feeding Practices by Country Distribution

According to this data, Côte d'Ivoire reported the highest proportion of appropriate feeding practices, with 97.32% (2861) women adhering to the recommended practices. Burkina Faso also demonstrated a high level of appropriate feeding practices, with 94.83% (3345) women. Similarly, Ghana and Kenya showed relatively good appropriate feeding practices, with 97.32% (2786) and 94.83% (5401) women, respectively.

Lesotho exhibited a lower percentage of appropriate feeding practices, with only 51.80% (735 women) following the recommended practices, while a substantial number, 602.44 women, engaged in inappropriate feeding behaviors. Madagascar demonstrated better adherence to appropriate feeding practices, with 816.45 women (approximately 24%) following the recommended guidelines, compared to 2620.56 women practicing inappropriate feeding.

Mozambique and Tanzania also had considerable proportions of inappropriate practices, with 2602.88 women in Mozambique and 2905.01 women in Tanzania following inappropriate practices. See the results in Table 1.

**TABLE 1:** Complementary feeding practices among women with children aged 6–23 months in eight African countries.

Country	Frequency	Appropriate feeding practices	In appropriate feeding practices
Burkina Faso	3345	94.83	3208.96
Côte d'Ivoire	2861	97.32	2546.56
Ghana	2786	305.92	2256.50
Kenya	5401	511.88	4337.57
Lesotho	735	51.80	602.44
Madagascar	3449	816.45	2620.56
Mozambique	2579	74.01	2602.88
Tanzania	3079	184.80	2905.01

### 3.4 Individual Characteristics of Complementary Feeding Practices Among Women With Children Aged 6–23 Months in Eight African Countries

According to these findings, women who are married report significantly higher values for appropriate feeding practices (1801.8). In contrast, widowed and divorced women show lower values for appropriate feeding practices (21.7 and 120.0, respectively). Women with no education report significantly lower values for appropriate feeding practices (262.1). However, women with secondary education or higher report the highest values for appropriate feeding practices (1282.9).

Women who use social media report higher appropriate feeding practices (1822.5) compared to those who do not use social media (314.5). Women in the “rich” wealth category report the highest value for appropriate feeding practices (1281.2), followed by women in the middle wealth category (342.8), and women in the poor category (512.9).

Mothers aged 25–34 report the highest appropriate feeding practices (1036.7). Women who delivered at health facilities report higher values for appropriate feeding practices (1674.1) compared to those who delivered at home (462.9). Additionally, women with access to a smartphone report better appropriate feeding practices (1666.0) compared to those without access (470.9).

Women who consider the distance to the health facility to be a significant problem report lower values for appropriate feeding practices (496.7). Women in urban areas report better appropriate feeding practices (1193.5) compared to those in rural areas (1193.5).

Women whose household head is male report better appropriate feeding practices (1663.5) compared to those whose household head is female (473.5). Households with fewer than 3 members report higher appropriate feeding practices (1288.2), while households with more than 3 members show lower values (848.8).

Women with a birth interval of more than 2 years show better appropriate feeding practices (1135.2) compared to those with an interval of less than 2 years (1001.8). Women who are currently breastfeeding report significantly higher appropriate feeding practices (1960.8) compared to those who are not breastfeeding (176.1).

Male children are associated with higher feeding practices (1115.7) compared to female children (1021.2). Women who had 4 or more antenatal care (ANC) visits report better appropriate feeding practices (1692.6) compared to those who had fewer than 4 visits (444.3).

Women who had a postnatal care (PNC) check‐up report better appropriate feeding practices (1504.1) compared to those who did not (632.9). Children aged 12–17 months show the best feeding practices (794.8 for appropriate feeding). Working mothers report better appropriate feeding practices (1479.3) compared to non‐working mothers (657.6). The results are in Table 2.

**TABLE 2:** Individual characteristics of complementary feeding practices among women with children aged 6–23 Months in eight African countries.

Variable	Category	Appropriate feeding practices	In appropriate feeding practices
Marital status	Single	193.4	1808.4
	Married	1801.8	17,834.9
	Widowed	21.70	188.7
	Divorced	120.0	1248.41
Education status	no educated	262.1	6779.9
	primary	592.0	7164.8
	secondary & above	1282.9	7135.6
Social media use	No	314.5	7286.8
	Ye	1822.5	13,793.6
Wealth status	Poor	512.9	9727.9
	Middle	342.8	4165.1
	Rich	1281.2	7187.3
Age of mother	15–24	675.5	7799.4
	25–34	1036.7	9243.8
	35–49	424.6	4037.2
Place of delivery	Home	462.9	4684.2
	Health facility	1674.1	16,396.2
Smartphone availability	NO	470.9	7878.3
	Yes	1666.0	13,202.1
Distance to HF	A big problem	496.7	9442.5
	Not a big problem	1640.2	11,637.9
Residence	Rural	943.5	6534.9
	urban	1193.5	14,545.5
Sex of the HH/Head	Male	1663.5	16,612.3
	Female	473.5	4468.1
Number of HH/Members	< 3	1288.2	10,429.4
	> 3	848.8	10,651.1
Birth interval	< 2 year	1001.8	8241.8
	> 2 year	1135.2	12,838.6
Current breastfeeding	No	176.1	4646.7
	Yes	1960.8	16,433.8
Sex of child	Male	1115.7	10,341.2
	Female	1021.2	10,739.3
Number of ANC visits	< 4	444.3	5875.9
	> = 4	1692.6	15,204.6
PNC Check‐up	No	632.9	7761.0
	Yes	1504.1	13,319.4
Child's age in months	6–11	691.5	7393.1
	12–17	794.8	7198.1
	> 18	650.6	6489.2
Mom's work status	No	657.6	9147.2
	Yes	1479.3	11,933.2

### 3.5 Determinants of Complementary Feeding Practices Among Women With Children Aged 6–23 Months

**FIGURE 4:**
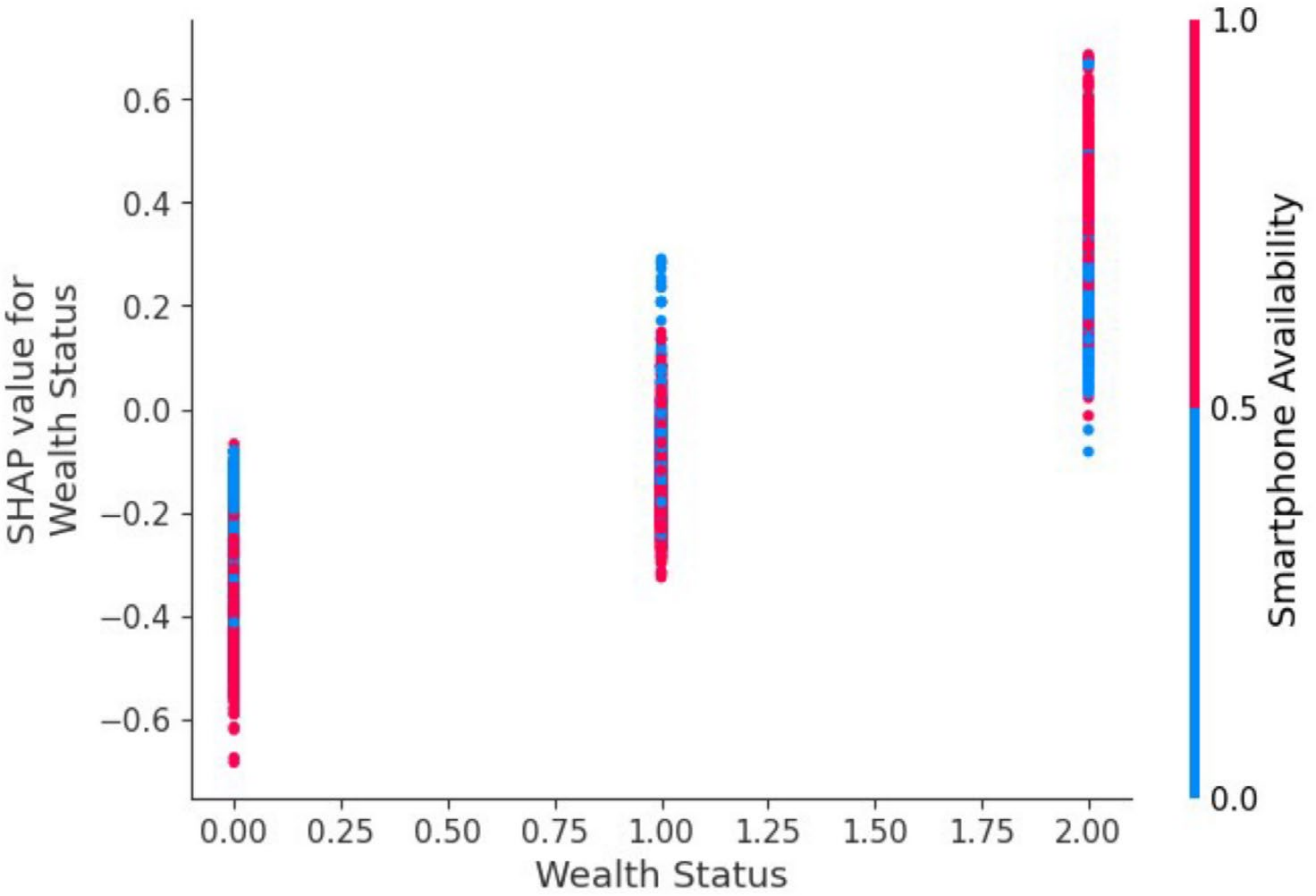
SHAP dependency plots.

**FIGURE 5:**
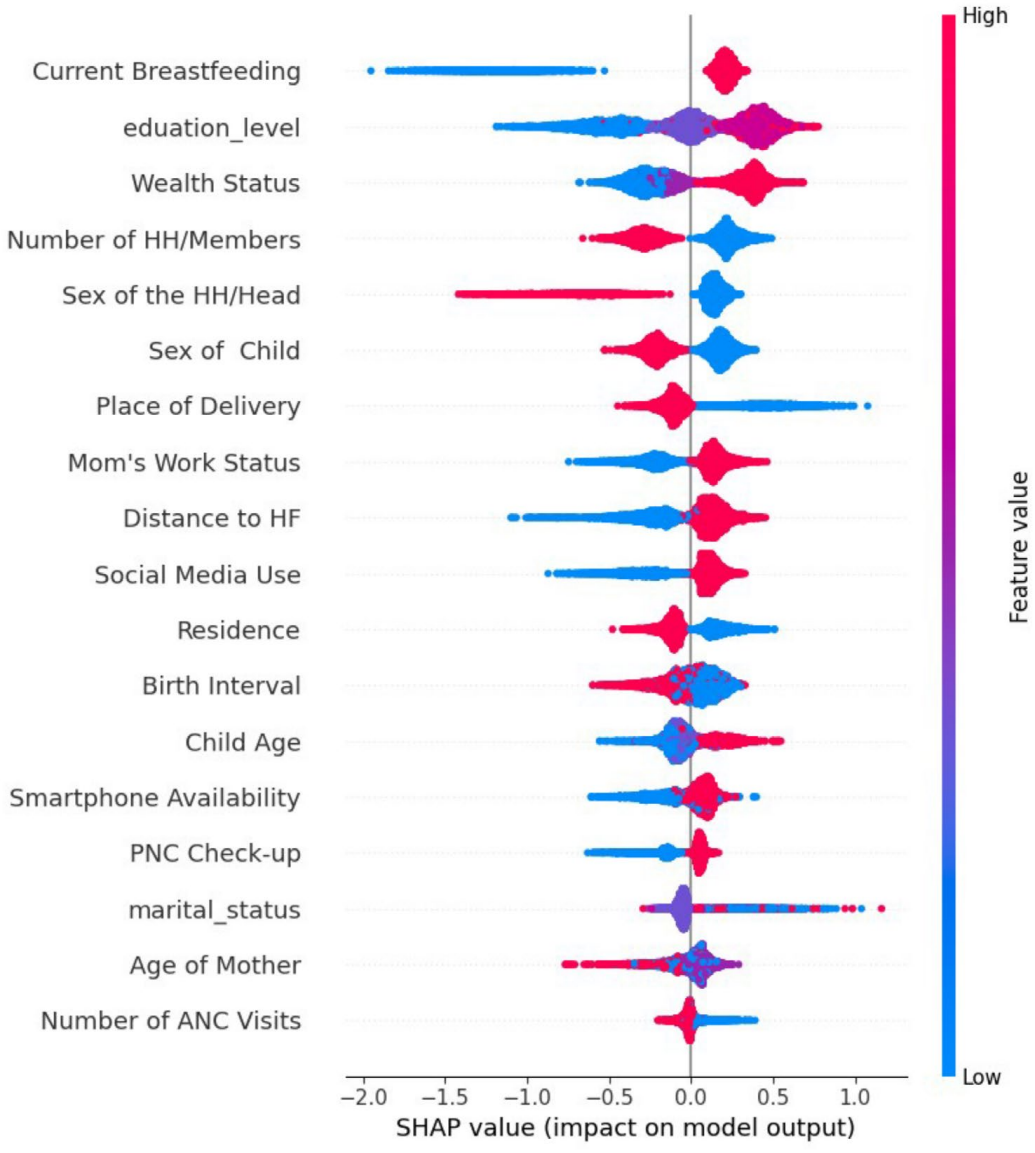
Predictors of appropriate complementary feeding practices among women with children aged 6–23 months.

Current breastfeeding, education status, wealth status, number of household members, sex of the household head, sex of the child, place of delivery, mother's work status, and distance to the health facility are the predictors for appropriate complementary feeding as identified by the random forest classifier. See the results in Figures 4 and 5.

### 3.6 Machine Learning Model Evaluation Metrics

**FIGURE 6:**
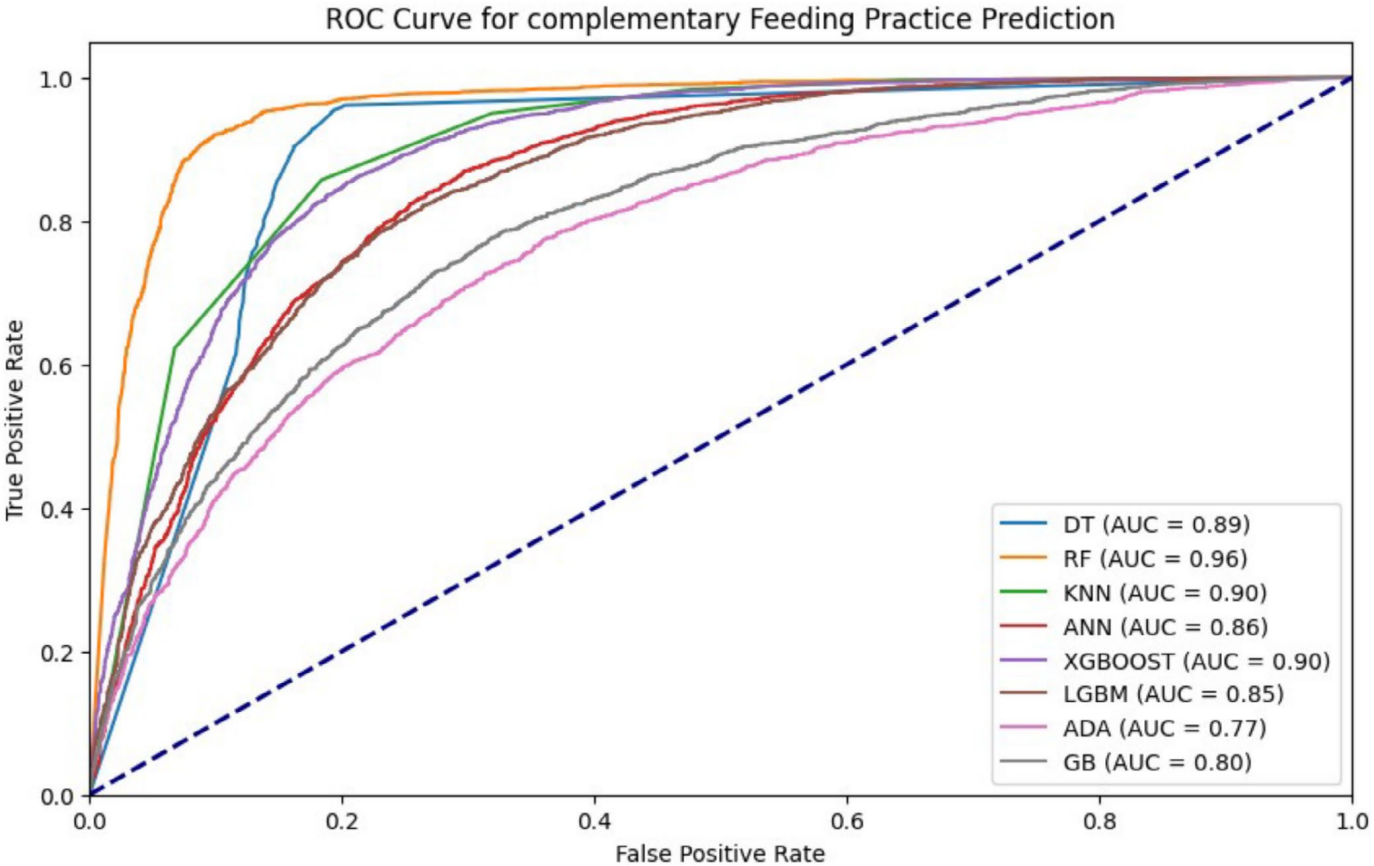
Model comparison by ROC curve for predicting complementary feeding practices.

Based on AUC, accuracy, F1 score, recall, and precision, Random Forest is the best‐performing model. The results are presented in Table 3, and Figure 6.

**TABLE 3:** Model comparison after SMOTE.

Model	AUC	Accuracy	F1 score	Recall	Precision
DT	89	88	87	86	88
RF	**96**	**91**	**91**	**92**	**90**
KNN	83	82	81	80	82
ANN	89	80	79	78	80
XGBoost	90	82	82	83	81
LGBM	85	77	76	75	77
ADA	77	70	69	68	70
GB	79	72	71	70	72

*Note:* Bold value shows the better performing algorithm.

## 4 Discussion

Appropriate complementary feeding practices in Sub‐Saharan Africa are a significant public health issue, as they affect the new generation of children throughout their lives. To increase appropriate feeding practices, strong and current evidence is required for policymakers and other relevant stakeholders. Therefore, we conducted this machine learning prediction study across a large study area, which includes eight Sub‐Saharan countries with recent DHS data, taking into account current interventions.

For this study, eight supervised machine learning models were used, namely DT, RF, KNN, ANN, XGBoost, LGBM, ADA, and GB. Among these, RF was the best‐performing model for predicting the determinants of appropriate complementary feeding practices, with an accuracy of 91% and an AUC of 96.

Random Forest is considered the best‐performing model in healthcare studies due to its ability to improve accuracy through ensemble learning, where multiple decision trees are combined to reduce overfitting. It effectively handles missing data, which is common in healthcare datasets, and which is valuable in healthcare to understand key factors influencing patient outcomes. Additionally, Random Forest can capture complex nonlinear relationships within healthcare data, unlike simpler models. Its versatility allows it to be applied to various healthcare problems, such as classification, regression, and outcome prediction, while providing high accuracy and robustness, making it a reliable choice for healthcare‐related predictions (Habib and Rahman 2021; Palacios‐Ariza et al. 2023; Santos et al. 2023; Tazin et al. 2021).

According to this finding, the appropriate complementary feeding practice for children aged 6 to 23 months in eight Sub‐Saharan countries is low, at only 9.2%. This is lower than a previous study in Africa, which reported 13.02%, Nigeria 10% (Ariyo et al. 2021), and the pooled proportion from a previous systematic study across different parts of Ethiopia is 21.77% (Dagne et al. 2022). According to our knowledge, there is limited research in Africa discussing this topic.

According to these machine learning SHAP findings, being a child of a current breastfeeding mother increases the likelihood of appropriate complementary feeding practices. The possible explanation for this finding could be that breastfeeding mothers are more likely to be engaged in child nutrition and health, as breastfeeding is often associated with a greater awareness of the importance of proper feeding practices. Additionally, breastfeeding may be part of a broader focus on child well‐being, encouraging mothers to continue with appropriate complementary feeding once the child reaches the appropriate age. Moreover, breastfeeding itself may contribute to better maternal–infant bonding, which could lead to improved care and feeding practices overall (Pérez‐Escamilla et al. 2023; Jebena and Tenagashaw 2022). According to the SHAP machine learning findings, women with secondary education or higher are more likely to engage in appropriate feeding practices. This is supported by earlier studies in Africa (Dagne et al. 2022; Acharya et al. 2019; Mulugeta et al. 2024; Wako et al. 2022).

The possible explanation could be that, due to increased knowledge of health, nutrition, and child development, educated women are better able to make healthier feeding choices. They also have better access to information about optimal feeding practices through books, the internet, and healthcare professionals, which enhances their ability to implement these practices effectively (Prasetyo et al. 2023).

Women with higher wealth status are more likely to adopt appropriate feeding practices, in line with previous studies in Africa (Masuke et al. 2021; Ahmed et al. 2022). The possible explanation could be that women with higher wealth status have better access to resources, education, healthcare, and improved socioeconomic conditions, which allow them to offer a more nutritious diet and obtain professional advice on child nutrition (Haque et al. 2024; Miller et al. 2017; Rekha et al. 2024).

In contrast, women with more than three household members are less likely to practice appropriate feeding practices, as supported by previous studies (Mekonen et al. 2024; Mulugeta et al. 2024; Al Mamun et al. 2022; Bwalya et al. 2023; Macharia et al. 2018). This is because women from larger families may struggle with appropriate feeding practices due to several factors. Resource constraints are common, as larger families often face financial challenges, spreading resources like nutritious food and time thin. Additionally, mothers may have less time and attention for each child, leading to suboptimal feeding practices. The caregiving burden in larger families can also affect the quality and frequency of complementary feeding (Ahishakiye et al. 2021; Dulal et al. 2022; Ikobah et al. 2023).

According to the SHAP machine learning findings, women in households with a male head are more likely to adopt appropriate feeding practices for their children. This could be attributed to factors such as increased financial resources, better access to healthcare, and support in decision‐making regarding child nutrition, which collectively contribute to improved feeding practices. This finding is supported by previous studies in Africa (Abate and Belachew 2017; Cunningham et al. 2021; Kukeba et al. 2021; Ziaei et al. 2015).

This finding also showed that women with female children are less likely to practice appropriate feeding. The reason could be that in various cultures, there is a tendency to prioritize the nutritional needs of male children over female children.

Women who have experience with home deliveries are less likely to practice appropriate complementary feeding for their children, according to common predictors identified in previous studies (Dagne et al. 2022; Areja et al. 2017; Ayu et al. 2024; Ickes et al. 2015; Tadesse et al. 2023). The possible explanation could be that women who give birth at home may have limited access to healthcare, leading to a lack of knowledge about proper complementary feeding. Home deliveries are often attended by unskilled individuals, resulting in a lack of professional guidance on infant care, including feeding (Weldegiorgis and Feyisa 2021).

Women with easy access to health facilities are more likely to practice appropriate feeding for their children. This fact is a common predictor, as identified in previous studies (Bwalya et al. 2023; Hashim et al. 2016).

Women who are active social media users are more likely to practice appropriate feeding for their children, in line with previous studies (Griauzde et al. 2020). This is why previous evidence has indicated that many mothers use social media and other platforms to find information on maternal and child health, including feeding practices for their children.

Moreover, women living in rural areas are less likely to practice appropriate feeding for their children in line with previous studies (Mulugeta et al. 2024; Bwalya et al. 2023; Yapa et al. 2020).

The machine learning model's SHAP results indicate that employed women are more likely to feed children appropriately, in keeping with earlier studies (Mulugeta et al. 2024; Bwalya et al. 2023; Yapa et al. 2020). The possible explanation could be that employed women frequently have better access to healthcare services and nutrition education (Soetan and Obiyan 2019). Employment can also increase a woman's decision‐making power and autonomy within the household (Zegeye et al. 2023). According to these findings, as the child's age increases, mothers are more likely to practice appropriate feeding, as supported by previous studies (Dagne et al. 2022; Ahmed et al. 2022; Bedada Damtie et al. 2020; Pesch et al. 2020).

Women who have smartphones are more likely to practice appropriate complementary feeding compared to those who do not. The possible explanation could be increased access to health information via smartphone use, reminders, and easy access to teleconsultations about nutrition for their children with nutritionists and other experts (Griauzde et al. 2020).

Women who have received postnatal care are more likely to practice appropriate complementary feeding, as supported by previous evidence (Dagne et al. 2022; Acharya et al. 2019; Mulugeta et al. 2024). A possible explanation is that increased awareness and knowledge about their child's feeding lead mothers to focus more on this aspect during postnatal care, applying health professionals' recommendations more actively than before (Acharya et al. 2019). Additionally, postnatal care includes monitoring the baby's growth and development, which further supports appropriate feeding practices (Dagne et al. 2022).

According to this SHAP finding, women who had fewer than three antenatal care visits are more likely to have poor practices of appropriate complementary feeding compared to those who had more than three visits. This is supported by earlier studies in Africa (Dagne et al. 2022; Mulugeta et al. 2024; Ahmed et al. 2022; Rahman et al. 2022). The possible explanation is that fewer than three antenatal care visits result in missed health education, counseling on complementary feeding, and reduced access to healthcare support.

### 4.1 Strengths and Limitations of the Study

A key strength of this study is its use of a large and diverse dataset, which enhances the generalizability of the findings across a broader geographic region. Additionally, machine learning models can handle complex patterns and relationships that may be overlooked by traditional statistical methods. However, the study has some limitations, including factors that can affect prediction accuracy, such as self‐reported data, recall bias, and missing information. Furthermore, the cross‐sectional nature of the data makes it challenging to establish causal relationships.

### 4.2 Conclusion and Recommendations

In conclusion, the Random Forest model was effectively used to identify key determinants, revealing that appropriate complementary feeding practices are low. To improve this, we recommend enhancing community‐based nutrition and reproductive health education, providing economic support for the poor, increasing access to healthcare facilities, and creating better opportunities to access smartphones and other social media platforms.

## Author Contributions

N.D.B., W.T. Methodology: H.T.E., M.G., T.A. Software: F.S.H. Validation: D.T.G., Y.T.A. Formal Analysis: T.Z.Y. Investigation: W.T., H.K.N. Resources: H.K.N. Data Curation: T.A., M.G. Writing Original Draft: N.D.B., T.Z.Y. Writing Review and Editing: H.T.E., D.T.G., Y.T.A. Visualization: F.S.H. Supervision: W.T., H.K.N. Project Administration: N.D.B. Funding Acquisition: W.T., H.K.N.

## Ethics Statement

Ethics approval for the study was obtained from the Measure of DHS (Demographic and Health Surveys) by submitting an online request.

## Consent

The authors have nothing to report.

## Conflicts of Interest

The authors declare no conflicts of interest.

## Data Availability

The datasets analyzed in the current study are available in the public domain through the Measure DHS website (www.measuredhs.com).
